# Interspecies co-feeding transmission of Powassan virus between a native tick, *Ixodes scapularis*, and the invasive East Asian tick, *Haemaphysalis longicornis*

**DOI:** 10.1186/s13071-024-06335-0

**Published:** 2024-06-15

**Authors:** Clemence Obellianne, Parker D. Norman, Eliane Esteves, Meghan E. Hermance

**Affiliations:** Department of Microbiology and Immunology, Frederick P. Whiddon College of Medicine, University of South Alabama, Mobile, AL USA

**Keywords:** Powassan virus, *Haemaphysalis longicornis*, Co-feeding transmission, Nonviremic transmission

## Abstract

**Background:**

Powassan virus, a North American tick-borne flavivirus, can cause severe neuroinvasive disease in humans. While *Ixodes scapularis* are the primary vectors of Powassan virus lineage II (POWV II), also known as deer tick virus, recent laboratory vector competence studies showed that other genera of ticks can horizontally and vertically transmit POWV II. One such tick is the *Haemaphysalis longicornis*, an invasive species from East Asia that recently established populations in the eastern USA and already shares overlapping geographic range with native vector species such as *I. scapularis*. Reports of invasive *H. longicornis* feeding concurrently with native *I. scapularis* on multiple sampled hosts highlight the potential for interspecies co-feeding transmission of POWV II. Given the absence of a clearly defined vertebrate reservoir host for POWV II, it is possible that this virus is sustained in transmission foci via nonviremic transmission between ticks co-feeding on the same vertebrate host. The objective of this study was to evaluate whether uninfected *H. longicornis* co-feeding in close proximity to POWV II-infected *I. scapularis* can acquire POWV independent of host viremia.

**Methods:**

Using an in vivo tick transmission model, *I. scapularis* females infected with POWV II (“donors”) were co-fed on mice with uninfected *H. longicornis* larvae and nymphs (“recipients”). The donor and recipient ticks were infested on mice in various sequences, and mouse infection status was monitored by temporal screening of blood for POWV II RNA via quantitative reverse transcription polymerase chain reaction (q-RT-PCR).

**Results:**

The prevalence of POWV II RNA was highest in recipient *H. longicornis* that fed on viremic mice. However, nonviremic mice were also able to support co-feeding transmission of POWV, as demonstrated by the detection of viral RNA in multiple *H. longicornis* dispersed across different mice. Detection of viral RNA at the skin site of tick feeding but not at distal skin sites indicates that a localized skin infection facilitates transmission of POWV between donor and recipient ticks co-feeding in close proximity.

**Conclusions:**

This is the first report examining transmission of POWV between co-feeding ticks. Against the backdrop of multiple unknowns related to POWV ecology, findings from this study provide insight on possible mechanisms by which POWV could be maintained in nature.

**Graphical Abstract:**

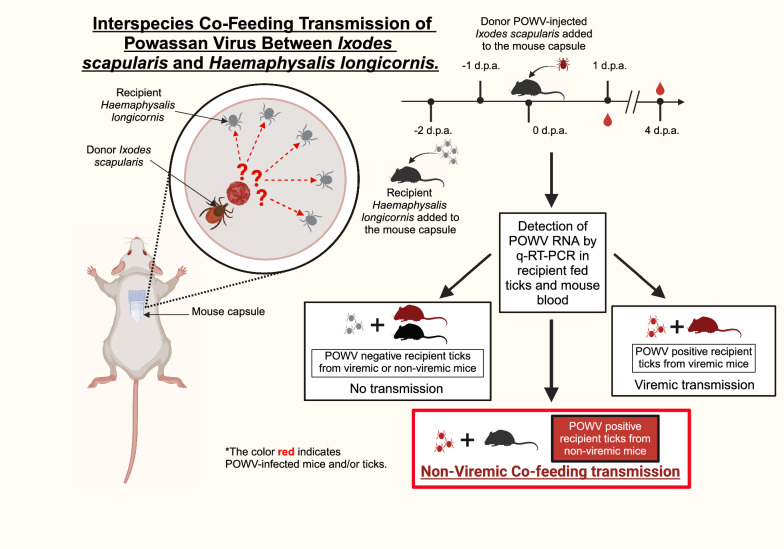

**Supplementary Information:**

The online version contains supplementary material available at 10.1186/s13071-024-06335-0.

## Background

Powassan virus (POWV) is a member of the *Orthoflavivirus* genus, belonging to the Flaviviridae family. POWV is primarily identified in the northeastern and Great Lakes regions of the USA, Southern Canada, and the Russian Far East. This zoonotic virus is transmitted to humans by several *Ixodes* species ticks [1–3], and it is the only tick-borne flavivirus endemic to North America. POWV was first identified as a human pathogen in 1958 in Powassan, Ontario when the virus was isolated from the brain tissue of a child who succumbed to encephalitis [4]. Initial clinical signs of POWV disease in humans include fever, headache, vomiting, and weakness. POWV infections can also progress to an altered mental state, encephalitis, seizures, paralysis, and coma [4–7]. The fatality rate for POWV cases is estimated to be 12.5–17.9% [8], with more than half of survivors experiencing long-term neurological sequelae [2]. There appears to be an increase in human cases of POWV in the USA in recent decades [3, 8, 9]. This rise could potentially be attributed to heightened surveillance of ticks and tick-borne diseases, increased clinician awareness and improved diagnostics, or the expansion of *Ixodes scapularis* populations [3, 10].

Following its initial identification, POWV was shown to be maintained in nature through transmission cycles involving *Ixodes* species ticks and mammalian hosts [11, 12]. POWV exhibits two genetic lineages: POWV lineage I (POWV I) and POWV lineage II (POWV II), also recognized as deer tick virus. Although serologically indistinguishable, these two POWV lineages share 84% and 93–94% nucleotide and amino acid sequence identity, respectively [13, 14]. It is also becoming increasingly apparent that POWV lineages I and II are ecologically distinct. POWV I was initially thought to be sustained in enzootic cycles involving *Ixodes cookei* and groundhogs/mustelids [12, 15] or *Ixodes marxi* and squirrels [11]. POWV II is typically isolated from *Ixodes scapularis*, which tend to have a catholic host preference and frequently parasitize humans. While POWV II was originally thought to be maintained in an enzootic transmission cycle between human-biting *I. scapularis* and white-footed mice (*Peromyscus leucopus*) [1, 16], the virus has never been isolated from mice in the field. Furthermore, laboratory infection of *P. leucopus* with POWV resulted in no overt clinical disease [17], suggesting that although *P. leucopus* are frequently exposed to POWV, they are an unlikely reservoir host. Various studies have assessed POWV experimental infections in several animal species. One study involving groundhogs, striped skunk, and fox squirrels [18] and another focusing on snowshoe hares [19] found that none of these vertebrate hosts developed POWV I or II viremias greater than ~ 1000 PFU/mL (Plaque-Forming Unit/mL), which indicates that these vertebrates are improbable reservoir hosts for POWV. A recent study used host-specific retrotransposon targeted real-time PCR on questing *I. scapularis* nymphs and demonstrated that there is a strong positive correlation between ticks feeding on shrews at a given site and the prevalence of POWV II infection in the ticks [20]. These data, together with the detection of POWV II RNA in the brain of one shrew, led the authors to conclude that shrews are a potential reservoir host for POWV II [20]; however, laboratory infection of shrews with POWV, as well as transmission studies with ticks, are needed to fully assess the reservoir host capacity of shrews.

In field studies, POWV I and POWV II have been detected in alternate hard-tick species (e.g., *Dermacentor andersoni*, *Dermacentor variabilis*) other than the main *Ixodes* vector species outlined above [21, 22]. Furthermore, recent laboratory studies demonstrate that *D. variabilis*, *H. longicornis* and *Amblyomma americanum* are competent vectors for POWV II [23, 24]. These findings suggest that the transmission cycles of POWV I and POWV II could be more complex than the single tick vector species/single reservoir host paradigm [25].

*Haemaphysalis longicornis* is an ixodid tick native to East and Southeast Asia and eastern Russia that has become established in Australia, New Zealand, several Pacific Island nations, and most recently, the USA. This tick species was first documented outside of a US port of entry when several *H. longicornis* were collected in Staten Island, New York in 2014–2015 [26]. Since its initial detection in the USA, established populations of *H. longicornis* have been documented in 19 states along the East Coast and Appalachia, with the most recent detection reported in Georgia in September 2022 [27]. The first record of *H. longicornis* biting a human in the USA was reported in June 2018 [28], indicating that populations of this tick species are already present in outdoor areas frequented by humans. In recent years, several studies have evaluated the ability of *H. longicornis* to acquire, maintain, and transmit tick-borne pathogens endemic to North America, ultimately demonstrating through laboratory studies that this tick is a competent vector for *Rickettsia rickettsii* and POWV II [24, 29]. This tick’s ability to transovarially transmit Heartland virus was also reported [30].

*Haemaphysalis longicornis* demonstrates adaptability to a broad range of climates in both its native and invasive ranges, including seasonal climates. Ecological niche modeling predicting the potential spread of *H. longicornis* in North America has identified several regions beyond the Northeast and East Coast as suitable for sustaining populations of *H. longicornis*. These regions include the Southeast, the Midwest, the California coast, the Pacific Northwest, and southeastern Canada [31–33]. Both the current distribution of *H. longicornis* (eastern USA and Appalachia), as well as the tick’s predicted geographic range expansion, overlap with the distribution of native tick species (e.g., *I. scapularis*, *D. variabilis*, *A. americanum*, etc.) that are competent vectors for POWV II.

In its native geographic range, both parthenogenetic and bisexual strains of *H. longicornis* have been observed; however, the North American populations of this invasive tick appear to reproduce exclusively by parthenogenesis [34]. *Haemaphysalis longicornis* is characterized as an aggressive biter and a host generalist. In its native range, it feeds on mammalian, avian, and human hosts [34]. Since its recent establishment in North America, this tick species has been observed feeding on a diverse array of hosts. Documented instances include various domestic animals and wildlife, ranging from rodent species to dogs and white-tailed deer [27, 35]. Interestingly, field surveillance studies conducted on Staten Island, New York detected *H. longicornis* feeding concurrently with at least one native tick species (*A. americanum* or *I. scapularis*) on 78% of sampled hosts [36, 37]. *Haemaphysalis longicornis* was found co-feeding on the same host body region alongside *I. scapularis* on 81.3% of white-tailed deer, 95.2% of opossums, 46.2% of raccoons, 50% of striped skunks, 50% of marmots, and 60% of feral cats; however, exact proximities between the co-feeding *H. longicornis* and *I. scapularis* were not reported [36].

While horizontal and vertical transmission are the most commonly evaluated routes of tick-borne virus transmission, tick-borne viruses can also be transmitted between co-feeding ticks. Co-feeding transmission occurs when an infected tick transmits virus to an uninfected tick while simultaneously feeding on the same host, often in close proximity on the skin. The phenomenon of co-feeding transmission (i.e., nonviremic transmission) was first described in the late 1980s when uninfected ticks, co-feeding on the same host with Thogoto virus-infected ticks, acquired virus even in the absence of host viremia [38]. Prior to this work by Jones et al., it was believed that an obvious viremia and systemic infection was necessary for ticks to acquire virus during blood feeding; however, Jones’ work was paradigm shifting in that it demonstrated that tick-to-tick transmission of virus can occur via nonviremic hosts. To date, no reservoir host has been clearly defined for POWV II; therefore, it is possible that POWV II may be sustained in natural transmission cycles via nonviremic transmission between co-feeding ticks [39], as has been shown for tick-borne encephalitis virus (TBEV) transmission in Europe [25].

With the many unknowns of POWV ecology and the yet to be determined role of invasive *H. longicornis* in tick-borne virus transmission cycles in North America, the objective of this study was to investigate whether and to what extent nonviremic co-feeding transmission of POWV II occurs when uninfected *H. longicornis* co-feed on the same host as an infected *I. scapularis*. By investigating co-feeding transmission of POWV II in the context of two different tick species with overlapping geographic distributions in portions of North America, the present study will provide us with important insight into a mechanism through which POWV could be maintained in nature.

## Methods

### Ethics and biosafety

All experiments involving virus-infected and mock-infected ticks fed on mice were conducted within a dedicated room of the animal biosafety level 3 (ABSL-3) facility. Infected ticks were handled and housed in arthropod containment level 3 (ACL-3) facilities. Bloodmeals were provided to uninfected ticks for colony maintenance by feeding ticks on guinea pigs in animal biosafety level 2 (ABSL-2) facilities. All biosafety level 3 (BSL-3), ABSL-3, and ACL-3 experiments were performed in accordance with protocols approved by the Institutional Biosafety Committee and the Institutional Animal Care and Use Committee (IACUC). Animal work was conducted using protocols approved by the University of South Alabama (USA) IACUC (protocol nos. 1619216, 2065516).

### Cells and virus

African green monkey kidney (VeroE6) cells were maintained in Dulbecco’s Modified Eagle Medium (DMEM, Genesee Scientific) with 10% fetal bovine serum (FBS) and 1% penicillin/streptomycin. Cells were cultured in an incubator set to 37 °C and 5% CO_2_ and used to generate virus stock. The Spooner strain of Powassan virus lineage II (POWV II) was acquired from the World Reference Center for Emerging Viruses and Arboviruses at the University of Texas Medical Branch. The stock had previously been passaged once on suckling mouse brains and five times on Vero cells. It was then passaged once on VeroE6 cells. The stock virus titer was determined by focus forming immunoassay as previously described [40].

### Ticks and animals

#### Ticks

A pathogen-free colony of *H. longicornis* was maintained by feeding ticks on Hartley guinea pigs under ACL-2 conditions. This colony originated from ticks collected in New York state in 2018. This lineage of *H. longicornis* has been propagated under laboratory conditions for seven generations without supplementation. Male and female *I. scapularis* were provided by Oklahoma State University. This colony originated from engorged females collected in Stillwater, Oklahoma in 1991. All tick vials were stored at 21 °C with 90–95% relative humidity. The photoperiod of the room was set on a 16:8 h light:dark cycle.

#### Mice

The 5-week-old male and female BALB/c mice were purchased from The Jackson Laboratory (Bar Harbor, ME). Mice were allowed to acclimate to the environment for a minimum of 5 days before the commencement of the experiments, at which point they were 6–6.5-weeks-old. During the study, mice were individually housed in ventilated cage systems maintained in a 12:12 h light:dark environment. Room humidity and temperature were closely regulated for the caging environments. Food and water were provided to mice ad libitum.

#### Tick feeding capsules used for tick infestations on mice

Mice were randomly assigned to infection or mock groups 1 day before tick infestation, and one tick containment capsule was attached per mouse. Capsules were fashioned from 2 mL cryotubes. The base of each tube was cut to leave approximately 3 mm of remaining tube below the screw-cap lid. The tops of the lids were cut to allow for an opening. The capsule was then inserted in the middle of a round piece of elastic adhesive bandage. Mice were anesthetized with isoflurane anesthesia to effect for the capsule placement procedures. The dorsum and lateral sides of each mouse were shaved with an electric razor and the capsule adhered to the mouse skin using Kamar livestock adhesive (Kamar Inc., Steamboat Springs, CO). At this point, mice were individually housed in micro-isolator cages. On the day of tick infestation, ticks were placed inside the capsules and a piece of fine mesh fabric was placed under the lid to allow for both tick containment and air exchange. Capsule lids were secured using masking tape. Capsule integrity was checked daily throughout tick infestation. Capsules were reinforced with adhesive and bandages as needed.

### RNA extractions from tick and mouse tissues

Pools of up to five fed recipient *H. longicornis* larvae, individual fed recipient *H. longicornis* nymphs, fed donor *I. scapularis* females, and mouse skin biopsies were homogenized with 3 mm sterile stainless-steel beads on a Qiagen Tissue Lyser II at 30 Hz for 3 min. These homogenized tissue samples as well as mouse blood were all stored in TRIzol reagent. A hybrid of TRIzol and Qiagen RNeasy Mini Kit protocols was used to perform RNA extractions. We have previously demonstrated that this hybrid protocol inactivates virus and produces high-quality RNA [24, 41]. A Nanodrop One Spectrophotometer (Thermo Fisher Scientific) was used to determine total RNA quantity and purity.

### Detection of viral RNA by q-RT-PCR

Absolute quantification of POWV II RNA quantities in tick and mouse samples was determined by quantitative reverse transcription real-time PCR (q-RT-PCR) as previously described [42]. Viral RNA quantities are expressed on a Log10 scale as the number of POWV II NS5 gene fragment copies per ng of RNA after normalization to a standard curve produced using serial tenfold dilutions of a 464-base pair POWV II NS5 gene fragment to estimate viral burden. q-RT-PCR was performed using forward (5′—GATCATGAGAGCGGTGAGTGACT—3′) and reverse (5′ –GGATCTCACCTTTGCTATGAATTCA—3′) primers and a probe (/56-FAM/TGAGCACCTTCACAGCCGAGCCAG/36-TAMSp/) specific to POWV II NS5 gene, as previously described [43]. When performing the assay, 1 µL of RNA was added to the appropriate wells of a 96-well PCR plate and samples were run in triplicate; 10 µM dilutions of the forward and reverse primers and probe were used with reagents from the iTaq Universal SYBR Green One-Step Kit (BioRad). The total reaction volume was 20 µL per well. Plates were sealed and run on a LightCycler^®^ 480 II PCR System (Roche) at the following cycle settings: 10 min at 50 °C, 1 min at 95 °C, 10 s at 95 °C, and 30 s at 60 °C for 45 cycles.

### Microinjection of female *I. scapularis* with POWV II

Infection via microinjection of female *I. scapularis* was achieved by injecting 292 nL, containing 1000 focus-forming units (FFU) of POWV II stock, into the immobilized tick’s anal aperture using glass microneedles, a digitally operated microinjector with a footswitch, and a dissecting microscope as previously described [24]. For the mock-infected groups, an equivalent volume of DMEM media was injected. Glass microneedles were made using a micropipette puller (Sutter Instrument) and glass capillaries (World Precision Instruments). Each capillary had an internal diameter of 0.530 mm and an outer diameter of 1.14 mm; these microneedles were pulled such that the tip had a smaller diameter than that of the tick anal pore. POWV II-injected *I. scapularis* were housed in ACL-3 facilities and were monitored for mortality twice daily for 4 days after microinjection.

### In vivo tick co-feeding experiments

The female *I. scapularis* microinjected with POWV II are defined as “donor” ticks in this study. For all in vivo experiments, each mouse was infested with a single microinjected female *I. scapularis* and a single naïve male *I. scapularis*. Microinjected *I. scapularis* females were infested on mice at 25–27 days post-microinjection. *Haemaphysalis longicornis* larvae and nymphs from the pathogen-free tick colony are defined as “recipient” ticks in this study. For all in vivo experiments, each mouse was infested with up to 53 *H. longicornis* larvae or up to 16 *H. longicornis* nymphs. The notation d.p.a. (days post-attachment) consistently indicates the attachment day of the donor *I. scapularis* female ticks.

### Overview of tick infestation schemes used for the in vivo experiments

**Fig. 1:**
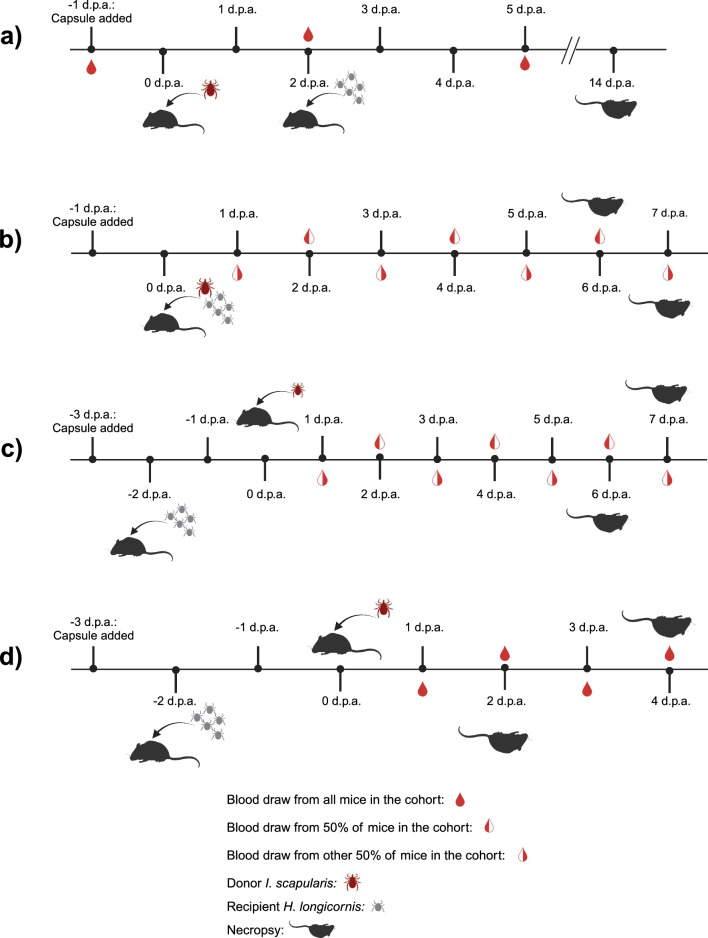
Tick infestation strategies used for the in vivo experiments. **a** “Staggered (donor first) tick infestation” timeline. Each mouse was infested with a single POWV II-injected donor *Ixodes scapularis* at 0 d.p.a. At 2 d.p.a., each mouse was then infested with recipient *Haemaphysalis longicornis* nymphs. Submandibular blood was collected from each mouse at −1, 2, and 5 d.p.a. Mice were euthanized at 14 d.p.a. or earlier if they met humane endpoints. **b **“Simultaneous donor and recipient tick infestation” timeline. Each mouse was infested with a single POWV II-injected donor *I. scapularis* and recipient *H. longicornis* nymphs at the same time (0 d.p.a.). Submandibular blood was collected every other day from each mouse. Mice were euthanized 2–3 days after completion of *H. longicornis* feeding for mice that did not reach humane endpoints. **c** “Staggered (recipient first) tick infestation” timeline. At −2 d.p.a., each mouse was infested with recipient *H. longicornis* larvae or nymphs, and 2 days later (0 d.p.a.), each mouse was then infested with a single POWV II-injected donor *I. scapularis*. Submandibular blood was collected every other day from each mouse. Mice were euthanized 2–3 days after completion of *H. longicornis* feeding for mice that did not reach humane endpoints. **d** “Staggered (recipient first) tick infestation with daily bleeds” and 2 or 4 d.p.a. necropsy timeline. At −2 d.p.a., each mouse was infested with recipient *H. longicornis* larvae or nymphs, and 2 days later (0 d.p.a.), each mouse was then infested with a single POWV II-injected donor *I. scapularis*. Submandibular blood was collected daily from each mouse until euthanasia at 2 or 4 d.p.a. *d.p.a.* days post-attachment, consistently indicates the attachment day of the donor *I. scapularis* female ticks, *POWV II* Powassan virus lineage II.

A staggered tick infestation scheme [“Staggered (donor first) tick infestation”] was used for the original in vivo experiment whereby mice were infested with donor *I. scapularis* first. Then, at 2 d.p.a. (relative to the *I. scapularis* donors), mice were infested with recipient *H. longicornis* nymphs (Fig. 1a). A simultaneous tick infestation scheme (“Simultaneous donor and recipient tick infestation”) was used for a subsequent in vivo experiment whereby mice were infested by donor *I. scapularis* and recipient *H. longicornis* nymphs at the same time (0 d.p.a.) (Fig. 1b). Finally, a flipped version of the staggered tick infestation scheme [“Staggered (recipient first) tick infestation”] was implemented whereby mice were infested by recipient *H. longicornis* larvae or nymphs first (−2 d.p.a.), and then by donor *I. scapularis* (0 d.p.a.) (Fig. 1c, d). This tick infestation scheme was repeated in three different cohorts of mice, each assessed separately over the course of approximately 1 year.

### Daily mouse clinical observations and weights

Mice were anesthetized at times of tick placement, capsule repair, tick removal, and submandibular bleed using isoflurane anesthesia. All mice were observed twice daily during the study period through the use of a clinical score chart that assesses weight loss, appearance (e.g., reduced grooming, ruffled coat, ocular/nasal discharge, hunched posture, etc.), neurologic disease (e.g., weak grip, paresis, ataxia, tremors, head tilt, partial paralysis, seizures, etc.), and behavior (e.g., subdued when stimulated, unresponsive). Mice that reached any of the following humane endpoints were immediately euthanized: > 20% weight loss, paralysis, seizures, gasping respiration, hemorrhage, prostrate, or unresponsive. Euthanasia occurred via isoflurane overdose followed by terminal cardiac puncture and cervical dislocation. For the original in vivo experiment [“Staggered (donor first) tick infestation, Fig. 1a], mice were euthanized either at 14 d.p.a. or earlier (7–11 d.p.a.) when they met humane endpoints. For all other in vivo experiments, euthanasia was performed via the same method 2–3 days after completion of *H. longicornis* feeding.

Tick-infested mice were also checked daily during this clinical observation for tick attachment status. Attachment of the female *I. scapularis* “donors” was monitored two to three times a day for the first 2 days. Adult female tick attachment day (0 d.p.a.) was carefully recorded. Mice were removed from the study if their corresponding donor *I. scapularis* failed to attach. A naïve *I. scapularis* male was added to each capsule to facilitate the female *I. scapularis* attachment, although it is likely that *I. scapularis* females had already mated prior to infestation on the host as they were co-housed with *I. scapularis* males for ~ 1 week prior to feeding. Mice were checked daily for naïve *H. longicornis* “recipient” (larvae and nymphs) attachment. Engorged “recipients” were retrieved from the capsules after detaching from the skin. “Donor” ticks were collected upon reaching full engorgement and detaching from the host, or at the time of euthanasia, whichever occurred first. Blood was collected from each mouse via submandibular bleed. For the “Staggered (donor first) tick infestation” study, submandibular blood was collected at −1, 2, and 5 d.p.a. (Fig. 1a). For the “Simultaneous donor and recipient tick infestation” study (Fig. 1b) and the first “Staggered (recipient first) tick infestation” study (Fig. 1c), submandibular blood was collected from each mouse every other day after *I. scapularis* donor attachment. For the final “Staggered (recipient first) tick infestation with daily bleeds” study (Fig. 1d), submandibular blood was collected daily from each mouse after *I. scapularis* donor attachment and until euthanasia.

#### Necropsies

Necropsies of mock-infected and POWV-infected mice were conducted under ABSL-3 conditions. Terminal blood collected via cardiac bleed was equally divided into TRIzol Reagent storage and serum separator tubes. Brain, liver, kidney, spleen, testes, skin from the tick attachment site (inside the capsule), and skin from outside of the tick attachment site (~ 3 cm caudal to the capsule) were harvested from mice during necropsy (Fig. 3a), and 4-mm biopsy punch tools were used to harvest the skin samples. A portion of each tissue sample was stored in TRIzol Reagent and the other portion in 10% neutral-buffered formalin. Formalin was exchanged after a minimum of 24 h and was allowed a minimum of 72 h total contact time with tissues.

### Statistical analyses

For the comparison of the experimental groups, analysis of variance (ANOVA) followed by Tukey as a post-test was used; *p* ≤ 0.05 was considered statistically significant.

## Results

### Staggered (donor first) tick infestation

A total of 24 donor *I. scapularis* females were individually transferred to 24 mice. Then, at 2 days post-attachment (d.p.a.), each mouse was infested with seven to ten recipient *H. longicornis* nymphs (Fig. 1a). Submandibular blood was collected from each mouse at −1, 2, and 5 d.p.a. to assess mouse viremia status (Fig. 1a). In total, 2 of the media-injected control donor *I. scapularis* and 11 of the virus-injected donor *I. scapularis* engorged and fed to repletion. Mice showing mild signs of Powassan (POW) disease were euthanized at the scheduled time of 14 d.p.a., whereas other mice developed neuroinvasive POW disease and reached humane endpoints requiring euthanasia between 7 d.p.a. and 11 d.p.a. These findings demonstrate that our POWV-infected *I. scapularis* donors transmit infectious virus to the mice.

All recipient *H. longicornis* nymphs from these 13 remaining mice were individually screened for POWV II RNA by q-RT-PCR (Table 1). No viral RNA was detected in recipient *H. longicornis* that co-fed on mice infested with a media-injected donor *I. scapularis* (Table 1). POWV II RNA was detected in 59.4% of recipient *H. longicornis* that co-fed on mice infested with a virus-injected donor *I. scapularis* (Table 1). At 2 d.p.a. and/or 5 d.p.a., POWV II RNA was detected in the blood of 10 out of the 11 mice that were fed upon by the virus-injected donor *I. scapularis*. Therefore, it was not possible to determine whether the presence of viral RNA in recipient *H. longicornis* resulted from mouse viremia or co-feeding transmission of virus between donor and recipient ticks. Only one mouse had no detectable viral RNA in both blood samples collected at 2 d.p.a. and 5 d.p.a.; however, the lack of data regarding viremia status from other days during the tick infestation prevents any clear conclusion about whether this mouse supported *H. longicornis* acquisition of POWV II RNA via nonviremic co-feeding transmission.

**Table 1:** Rate of detection of POWV II RNA by q-RT-PCR in fed recipient *H. longicornis* from the “Staggered (donor first) tick infestation”

	POWV RNA detected in fed *H. longicornis* via q-RT-PCR % (#positive/total screened)	Range of positive ticks Log_10_(gene fragment copies/ng of RNA)	Median of positive ticks Log_10_(gene fragment copies/ng of RNA)	Median (all ticks) Log_10_(gene fragment copies/ng of RNA)
Naïve *H. longicornis* nymphs co-fed with POWV-injected *I. scapularis* female	59.4% (38/64)	0.999–4.393	1.492	1.152
Naïve *H. longicornis* nymphs co-fed with media-injected *I. scapularis* female	0% (0/10)	NA	NA	0

*POWV* Powassan virus, *RNA* ribonucleic acid, *q-RT-PCR* quantitative reverse transcription polymerase chain reaction, *H. longicornis* *Haemaphysalis longicornis*, *I. scapularis* *Ixodes scapularis*, *NA* not applicable

### Simultaneous donor and recipient tick infestation

In total, eight donor *I. scapularis* females were individually transferred to eight mice, and mice were each simultaneously infested with nine to ten recipient *H. longicornis* nymphs (Fig. 1b). Submandibular blood was collected every other day from each mouse to assess viremia status during tick co-feeding (Fig. 1b). All donor *I. scapularis* (media-injected and POWV II-injected) attached and fed. As expected, no viral RNA was detected in media-injected donor *I. scapularis*, whereas viral RNA was detected in 100% of virus-injected donor *I. scapularis* (Supplementary Fig. 1). All recipient *H. longicornis* nymphs were individually screened for POWV II RNA by q-RT-PCR upon detachment and completion of feeding (Table 2). No viral RNA was detected in recipient *H. longicornis* nymphs that co-fed on mice infested with a media-injected donor *I. scapularis*; however, POWV II RNA was detected in 44.4% of recipient *H. longicornis* nymphs that co-fed on mice infested with a virus-injected donor *I. scapularis* (Table 2). POWV II RNA was detected in the blood of mice corresponding to virus positive recipient *H. longicornis* nymphs on the same day or on a day before these recipient *H. longicornis* nymphs completed feeding. Therefore, similar to results obtained in the “Staggered (donor first) tick infestation” (Fig. 1a) described above, it is not possible to determine whether the presence of viral RNA in recipient *H. longicornis* resulted from mouse viremia or from co-feeding transmission of virus between the donor *I. scapularis* and recipient *H. longicornis*.

**Table 2:** Rate of detection of POWV II RNA by q-RT-PCR in fed recipient *H. longicornis* from the “Simultaneous donor and recipient tick infestation”

	POWV RNA detected in fed *H. longicornis* via q-RT-PCR % (#positive/total screened)	Range of positive ticks Log_10_(gene fragment copies/ng of RNA)	Median of positive ticks Log_10_(gene fragment copies/ng of RNA)	Median (all ticks) Log_10_(gene fragment copies/ng of RNA)
Naïve *H. longicornis* nymphs co-fed with POWV-injected *I. scapularis* female	44.4% (20/45)	1.073–3.557	1.558	0
Naïve *H. longicornis* nymphs co-fed with media-injected *I. scapularis* female	0% (0/12)	NA	NA	0

*POWV* Powassan virus, *RNA* ribonucleic acid, *q-RT-PCR* quantitative reverse transcription polymerase chain reaction, *H. longicornis* *Haemaphysalis longicornis*, *I. scapularis* *Ixodes scapularis*, *NA* not applicable

### Staggered (recipient first) tick infestation

In this tick infestation experiment, recipient *H. longicornis* were infested on mice prior to the donor *I. scapularis* in an effort to allow the recipient ticks to start feeding prior to the mouse developing viremia. Thus, at −2 d.p.a., 8 mice were each infested with 47–50 recipient *H. longicornis* larvae and another 8 mice were each infested with 9–10 recipient *H. longicornis* nymphs (Fig. 1c); 2 days later (0 d.p.a.), the mice were each infested with one donor *I. scapularis* female tick. Submandibular blood was collected every other day from each mouse to assess mouse viremia status (Fig. 1c), and 15 out of the 16 donor *I. scapularis* (media-injected and POWV II-injected) attached and fed. As expected, no POWV II viral RNA was detected in media-injected donor *I. scapularis*, whereas viral RNA was detected in 100% of POWV II-injected donor *I. scapularis* (Supplementary Fig. 1). Recipient *H. longicornis* were screened for POWV II RNA by q-RT-PCR (Table 3). No viral RNA was detected in recipient *H. longicornis* larvae or nymphs that co-fed on mice infested with a media-injected donor *I. scapularis*, whereas POWV II RNA was detected in 19.4% of recipient *H. longicornis* larvae (screened in pools) and 14.3% of recipient *H. longicornis* nymphs (screened individually) that co-fed on mice infested with a virus-injected donor *I. scapularis* (Table 3).

**Table 3:** Rate of detection of POWV II RNA by q-RT-PCR in fed recipient *H. longicornis* from the “Staggered (recipient first) tick infestation”

	POWV RNA detected in fed *H. longicornis* via q-RT-PCR % (#positive/total screened)	Range of positive ticks Log_10_(gene fragment copies/ng of RNA)	Median of positive ticks Log_10_(gene fragment copies/ng of RNA)	Median (all ticks) Log_10_(gene fragment copies/ng of RNA)
Naïve *H. longicornis* larvae co-fed with POWV-injected *I. scapularis* female	19.4%^a^ (7/36)	1.226–2.986	1.580	0
Naïve *H. longicornis* larvae co-fed with media-injected *I. scapularis* female	0% (0/14)	NA^a^	NA	0
Naïve *H. longicornis* nymphs co-fed with POWV-injected *I. scapularis* female	14.3% (6/42)	1.396–2.675	2.014	0
Naïve *H. longicornis* nymphs co-fed with media-injected *I. scapularis* female	0% (0/12)	NA	NA	0

*POWV* Powassan virus, *RNA* ribonucleic acid, *q-RT-PCR* quantitative reverse transcription polymerase chain reaction, *H. longicornis* *Haemaphysalis longicornis*, *I. scapularis* *Ixodes scapularis*, *NA* not applicable

^a^Rate of detection of POWV II RNA in larval pools

**Fig. 2:**
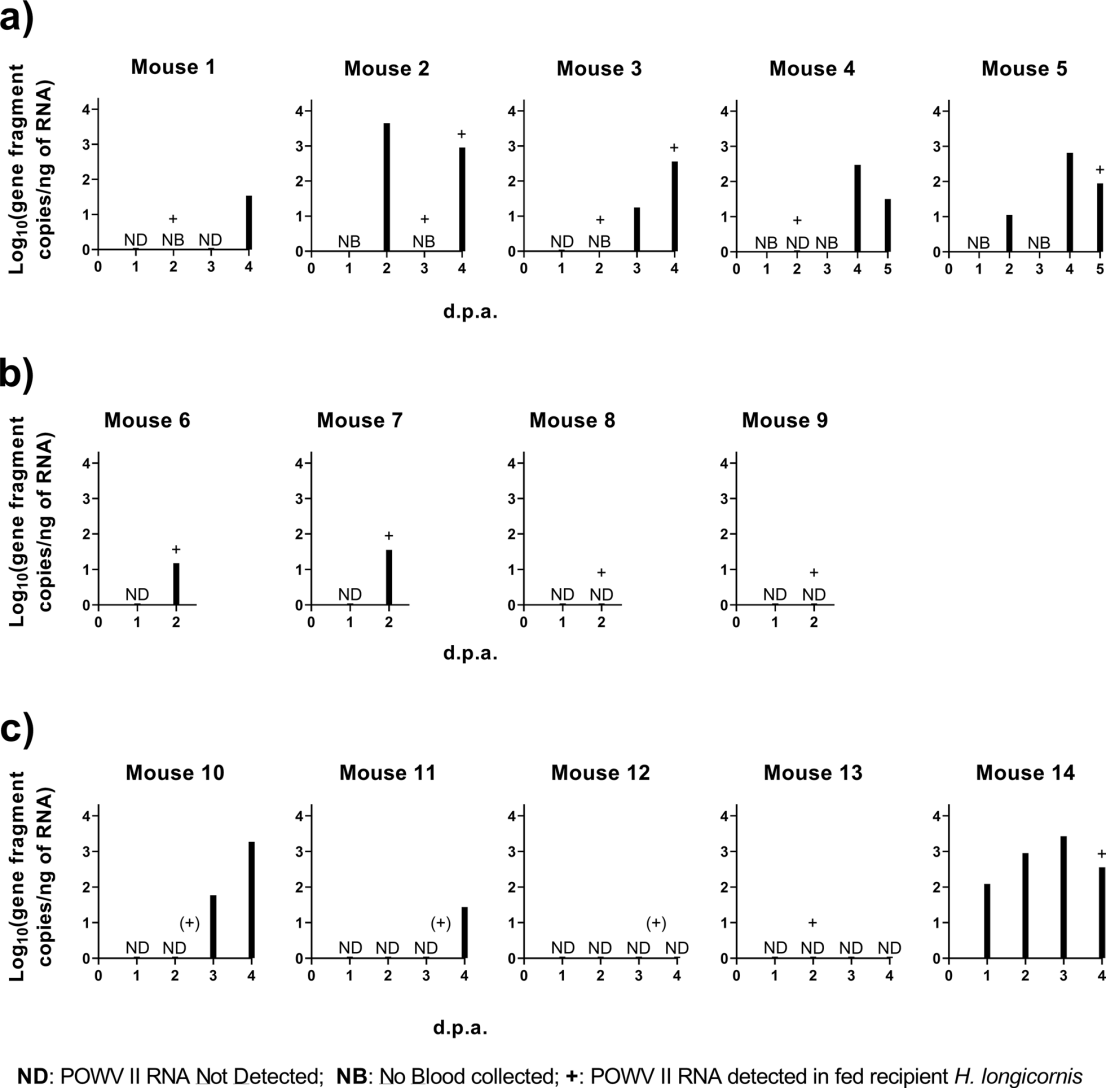
Detection of POWV II RNA in the blood of mice that yielded recipient *H. longicornis* positive for POWV II RNA. **a** Mice 1–5 are from the “Staggered (recipient first) tick infestation.” Each of these mice were bled every other day. **b** Mice 6–9 are from the “Staggered (recipient first) tick infestation with daily bleeds.” These mice were bled daily and euthanized at 2 d.p.a. **c** Mice 10–14 are from the “Staggered (recipient first) tick infestation with daily bleeds.” These mice were bled daily and euthanized at 4 d.p.a. POWV II RNA was detected in mouse blood and recipient *H. longicornis* samples via q-RT-PCR. Viral RNA quantities are expressed as the number of NS5 gene fragment copies per ng of RNA after normalization to a standard curve. Each bar represents the viral RNA quantity in the blood for that mouse at a specific time point. “ND” means that POWV II RNA was Not Detected in the blood. “NB” means that No Blood was collected on that day. The “+” symbol means that fed recipient *H. longicornis* screened positive for POWV II RNA at that timepoint. *d.p.a.* days post-attachment, consistently indicates the attachment day of the donor *I. scapularis* female ticks, *POWV II* Powassan virus lineage II, *RNA* ribonucleic acid, *q-RT-PCR* quantitative reverse transcription polymerase chain reaction.

Figure 2a shows the timeline of viremia for each of the five mice that yielded engorged *H. longicornis* recipients positive for POWV II RNA. The bar graphs show the temporal development of viremia as well as the days where virus-positive engorged *H. longicornis* recipients were collected (as indicated by the “+” symbol) from each mouse. For mouse 2, mouse 3, and mouse 5, virus-positive recipient *H. longicornis* were collected at timepoints when viral RNA was also detected in the blood on the same day or on the day prior. POWV II RNA was detected in five recipient *H. longicornis* nymphs that detached at 2 d.p.a. from mouse 4, but no POWV II RNA was detected in blood at 2 d.p.a. for this mouse (as shown in Fig. 2a with the “Not Detected” symbol), suggesting that nonviremic co-feeding transmission occurred. Similarly, viral RNA was detected in one pool of recipient *H. longicornis* larvae that detached from mouse 1 at 2 d.p.a., and although no blood sample was collected from mouse 1 at that timepoint, viral RNA was not detected in the blood samples collected the day before (1 d.p.a.) and the day after (3 d.p.a.) the pool of recipient *H. longicornis* larvae screened positive for POWV II RNA. These findings suggest that nonviremic co-feeding transmission is likely to have occurred for the virus-positive *H. longicornis* recipients collected from mouse 1. However, the lack of daily blood samples collected from each mouse in this experiment prevents a definitive conclusion regarding the mode of acquisition of POWV II RNA by these recipient *H. longicornis* nymphs.

### Staggered (recipient first) tick infestation with daily bleeds

To assess the viremia status of the vertebrate host with more granularity, a tick infestation experiment was designed in which recipient *H. longicornis* were infested on mice 2 days prior to the donor *I. scapularis*, each mouse was bled daily, and then mice were euthanized at either 2 d.p.a. or 4 d.p.a. (Fig. 1d). Here, 11 mice were each infested with 35–53 recipient *H. longicornis* larvae and another 13 mice were each infested with 12–16 recipient *H. longicornis* nymphs; 2 days later (0 d.p.a.), the mice were each infested with 1 donor *I. scapularis* female tick. All donor *I. scapularis* (media-injected and POWV II-injected) attached and fed. No viral RNA was detected in media-injected donor *I. scapularis*, but POWV II RNA was detected in 100% of virus-injected donor *I. scapularis* (Supplementary Fig. 1). All recipient *H. longicornis* were screened for POWV II RNA by q-RT-PCR (Table 4). No viral RNA was detected in recipient *H. longicornis* larvae or nymphs that co-fed on mice infested with a media-injected donor *I. scapularis*. However, in mice euthanized at 2 d.p.a. and infested with a virus-injected donor *I. scapularis*, POWV II RNA was detected in 50% of recipient *H. longicornis* larvae (screened in pools) and 12.5% of recipient *H. longicornis* nymphs (screened individually). On mice euthanized at 4 d.p.a. and infested with a virus-injected donor *I. scapularis*, POWV II RNA was detected in 100% of pooled *H. longicornis* larvae and 4.1% of individual *H. longicornis* nymphs (Table 4).

**Table 4:** Rate of detection of POWV II RNA by q-RT-PCR in fed recipient *H. longicornis* from the “Staggered (recipient first) tick infestation with daily bleeds”

	Necropsy day	POWV RNA detected in fed *H. longicornis* via q-RT-PCR% (#positive/total screened)	Range of positive ticks Log_10_(gene fragment copies/ng of RNA)	Median of positive ticks Log_10_(gene fragment copies/ng of RNA)	Median (all ticks) Log_10_(gene fragment copies/ng of RNA)
Naïve *H. longicornis* larvae co-fed with POWV-injected *I. scapularis* female	2 d.p.a.^a^	50% (2/4)^b^	1.038–1.592	1.315	0.124
Naïve *H. longicornis* larvae co-fed with media-injected *I. scapularis* female	2 d.p.a	All larvae dead and unfed	NA	NA	NA
Naïve *H. longicornis* nymphs co-fed with POWV-injected *I. scapularis* female	2 d.p.a	12.5% (4/32)	1.105–2.432	1.286	0.123
Naïve *H. longicornis* nymphs co-fed with media-injected *I. scapularis* female	2 d.p.a	0% (0/11)	NA	NA	0
Naïve *H. longicornis* larvae co-fed with POWV-injected *I. scapularis* female	4 d.p.a	100% (3/3)^b^	2.072–2.783	2.555	1.507
Naïve *H. longicornis* larvae co-fed with media-injected *I. scapularis* female	4 d.p.a	0% (0/2)^b^	NA	NA	0
Naïve *H. longicornis* nymphs co-fed with POWV-injected *I. scapularis* female	4 d.p.a	4.1% (2/49)	1.375–1.843	1.609	0
Naïve *H. longicornis* nymphs co-fed with media-injected *I. scapularis* female	4 d.p.a	0% (0/13)	NA	NA	0

*POWV* Powassan virus, *RNA* ribonucleic acid, *q-RT-PCR* quantitative reverse transcription polymerase chain reaction, *H. longicornis* *Haemaphysalis longicornis*, *I. scapularis* *Ixodes scapularis*, *NA* not applicable

^a^Days post-attachment of donor *Ixodes scapularis*

^b^Rate of detection of POWV II RNA in larval pools

Figure 2b, c represents the nine mice that yielded engorged *H. longicornis* recipients positive for POWV II RNA. Figure 2b, c bar graphs show the temporal development of viremia (assessed by daily blood samples collected from every mouse) relative to the days where virus-positive fed *H. longicornis* recipients were collected (as indicated by the “+” symbol) from each mouse. POWV II RNA was detected in recipient *H. longicornis* nymphs that detached at 2 d.p.a. from mouse 8, mouse 9, and mouse 13, but no viral RNA was detected in the blood of these mice at any timepoint. Recipient *H. longicornis* larvae detached from mouse 12 at 3 d.p.a. and 4 d.p.a. were combined into a single pool for POWV II RNA screening. Viral RNA was detected in this larval pool, but no viral RNA was detected in the blood of mouse 12 at any timepoint. Together, these findings clearly demonstrate that *H. longicornis* larvae and nymphs were capable of acquiring POWV II RNA from donor *I. scapularis* via nonviremic co-feeding transmission on mouse 8, mouse 9, mouse 12, and mouse 13 (Fig. 2b, c).

**Fig. 3:**
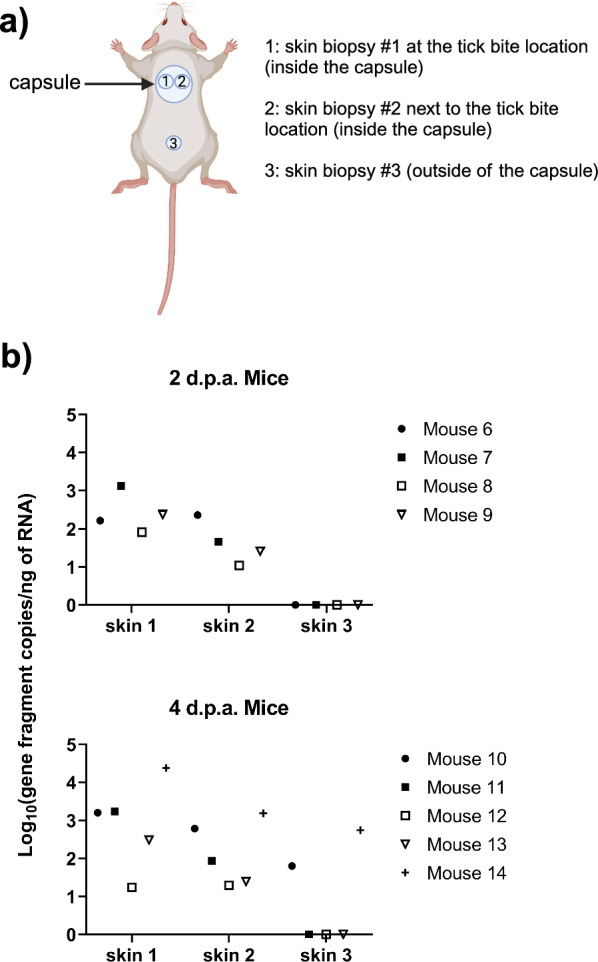
Detection of POWV II RNA in skin biopsies proximal and distal to the tick co-feeding site. **a** Location of skin biopsies #1, #2, and #3. **b** Mice 6–14 are from the “Staggered (recipient first) tick infestation with daily bleeds”. These mice were bled daily and euthanized at 2 d.p.a. or 4 d.p.a. POWV II RNA was detected in mouse skin biopsy samples via q-RT-PCR. Viral RNA quantities are expressed as the number of NS5 gene fragment copies per ng of RNA after normalization to a standard curve. Each point represents the viral RNA quantity in a skin biopsy for a specific mouse, as indicated in the legend. *d.p.a.* days post-attachment, consistently indicates the attachment day of the donor *I. scapularis* female ticks, *POWV II* Powassan virus lineage II, *RNA* ribonucleic acid, *q-RT-PCR* quantitative reverse transcription polymerase chain reaction.

For mice in the “Staggered (recipient first) tick infestations with daily bleeds” (Fig. 1d), skin biopsies collected proximal to the tick feeding site (from inside the tick feeding capsule) and skin biopsies collected distal to the tick feeding site (from outside the tick feeding capsule) were screened by q-RT-PCR for POWV II RNA (Fig. 3a). No POWV II RNA was detected in skin biopsy #3 for mice 6–9 and 11–13 (Fig. 3b). The blood of mouse 8, mouse 9, mouse 12, and mouse 13 was also negative for POWV II RNA at each day of tick feeding (Fig. 2b, c), so the absence of viral RNA in skin biopsy 3 provides additional evidence that there was no systemic infection in these mice. Furthermore, in the nonviremic mice, detection of viral RNA in skin from the tick feeding site (biopsies 1 and 2) but not at distal skin sites (biopsy 3) indicates that a localized skin infection may facilitate transmission of POWV II RNA between donor and recipient ticks co-feeding in close proximity.

## Discussion

Co-feeding transmission has been observed with various vector-borne pathogens, and its importance in natural transmission cycles of tick-borne encephalitis virus (TBEV), a close relative to POWV, was clearly demonstrated several decades ago [44–46]. TBEV primary vectors are *Ixodes ricinus* in Europe and *Ixodes persulcatus* in Fennoscandia, Russia, and Asia [47]. Nymphs and larvae of these two species are typically active in the same seasons and share the same rodent hosts, which enables intraspecies co-feeding transmission between juvenile tick stages [48]. In Europe, tick co-feeding transmission of TBEV plays an important role in the persistence of this virus within certain foci in nature [46]. Additionally, epidemiological modeling shows that co-feeding transmission alone (in the absence of vertical transmission or amplifying hosts) could be sufficient to sustain POWV in tick populations [39]. During nonviremic co-feeding transmission, an infected tick can transmit a virus directly to a naïve tick co-feeding on the same host (often in close proximity) without requiring the virus to be circulating in the blood of the vertebrate host. Therefore, co-feeding transmission can be highly efficient since the virus does not need to first replicate and disseminate within the vertebrate host to be transmitted between ticks, nor is a susceptible vertebrate host required for virus transmission via this mechanism. Labuda et al. showed that TBEV can be transmitted from infected to uninfected ticks feeding on certain virus-immune natural rodent hosts via nonviremic co-feeding transmission [44]. Despite these wild rodents being immunized against the virus, their neutralizing antibodies to TBEV did not prevent subsequent transmission of TBEV between co-feeding ticks [44]. Perhaps in North America, certain vertebrate hosts for *Ixodes* species ticks (e.g., *P. leucopus*) that are refractory to POWV infection and do not develop a patent viremia are indeed playing a role in sustaining POWV by serving as a transient bridge while the ticks serve as both the reservoirs as well as the vectors.

Under laboratory conditions, TBEV-infected adult *I. ricinus* co-fed with naïve *Rhipicephalus appendiculatus* or *Haemaphysalis inermis* nymphs, and TBEV-infected adult *Dermacentor reticulatus* co-fed with naïve *H. inermis* nymphs, all resulted in interspecies co-feeding transmission of TBEV [47, 48]. The present study also involved donor *Ixodes* species females that were infected with a flavivirus and co-fed with immature recipient ticks from a different genus. Data from this study demonstrate experimental interspecies co-feeding transmission of POWV II RNA between an adult virus-infected *I. scapularis* and naïve recipient *H. longicornis*, both in the presence and absence of host viremia. The rate of detection of POWV II RNA was highest in recipient *H. longicornis* that co-fed on viremic mice (Tables 1, 2). However, multiple recipient *H. longicornis* larvae and nymphs were able to acquire viral RNA while feeding concurrently and in close proximity to the infected *I. scapularis* donor with no viral RNA detected in the mouse blood. In the initial “Staggered (recipient first) tick infestation” (Fig. 1c), we showed co-feeding transmission of POWV II RNA from donor *I. scapularis* to recipient *H. longicornis* nymphs and larvae (Table 3, Fig. 2a). Although blood samples from mouse 1 and mouse 4 were not screened daily for POWV II RNA, the data suggest that these mice were nonviremic on days that the recipient *H. longicornis* screened positive for viral RNA (Fig. 2a). Nonviremic co-feeding transmission of viral RNA was then confirmed by the “Staggered (recipient first) tick infestation with daily bleeds” (Fig. 1d) via the detection of POWV II RNA in several recipient *H. longicornis* nymphs and larvae from mouse 8, mouse 9, mouse 12, and mouse 13 (Table 4) and the demonstration of the absence of viremia in these mice (Fig. 2b, c). In the “Staggered (recipient first) tick infestation” experiments, pathogen-free *H. longicornis* recipients were allowed to pre-feed on mice for 2 days prior to addition of the POWV-infected *I. scapularis* donors. Thus, it is possible that the presence of *H. longicornis* saliva already deposited at the tick co-feeding site facilitates transmission of virus, as previously demonstrated in studies showing that tick salivary gland extract facilitates POWV transmission and dissemination in the vertebrate host [41].

In the present study, POWV II RNA was detected in the skin from the tick feeding site (skin biopsies #1 and #2) but not at distal tick-free skin sites (skin biopsy #3) in the nonviremic mice (Fig. 3). These findings suggest that a localized skin infection and the spatiotemporal feeding proximity of the donor *I. scapularis* with the recipient *H. longicornis* supported nonviremic co-feeding transmission of POWV II RNA. Previous studies also highlighted the importance of ticks feeding in close proximity and localized virus infection at the site of tick attachment during the early stages of flavivirus transmission via tick co-feeding. When TBEV-infected and uninfected *I. ricinus* were infested on natural murine host species and either allowed to co-feed in close proximity inside the same capsule or retained in separate capsules and fed at a greater distance, TBEV was preferentially recruited to the tick-infested skin sites compared with tick-free skin, and TBEV transmission between co-feeding ticks was correlated with localized skin infection at the tick feeding sites [49]. Furthermore, co-feeding transmission of TBEV between infected and uninfected ticks was most efficient when ticks fed in close proximity but did still occur when ticks co-fed at a greater distance [49]. In nature, ticks often feed closely clustered together on their host. This is especially the case for many species of immature ticks that feed on rodents, whereby ~ 90% are commonly found on the ears, neck, and head of their murine hosts [50–52]. The present study evaluated tick co-feeding within a small (~ 1–1.5 cm) area, but it will be important for future studies to examine the outcome of co-feeding transmission of POWV when ticks feed at greater distances on the same vertebrate host. Additionally, while our data suggest that a localized skin infection plays an important role in the nonviremic co-feeding transmission of POWV, the mechanism underlying this phenomenon needs to be defined. Therefore, we have initiated studies investigating the mechanistic aspects of nonviremic co-feeding transmission.

Recent studies demonstrate that *D. variabilis*, *A. americanum*, and *H. longicornis* are competent vectors for POWV II under laboratory conditions [23, 24]. By detecting POWV II RNA in engorged *H. longicornis* that co-fed with POWV-infected *I. scapularis*, the present study provides evidence that multiple tick species, including invasive *H. longicornis*, could contribute to POWV maintenance in nature via interspecies co-feeding transmission; however, future investigations need to focus on detecting infectious virus in the recipient ticks to fully demonstrate virus transmission via tick co-feeding. Furthermore, all of the recipient *H. longicornis* in this study were processed as engorged larvae/nymphs, and as such, we did not screen recipient ticks post-molt for the presence of virus; however, previous studies have shown evidence of transstadial transmission of flavivirus occurring in *H. longicornis* [24, 53]. Despite these limitations, the present study clearly showed that interspecies tick co-feeding transmission of POWV II RNA occurs: (1) on viremic and nonviremic vertebrate hosts, (2) when various life stages of *H. longicornis* serve as the recipient, and (3) when donor and recipient ticks are infested on the host in different sequences. Perhaps most relevant to our current understanding of POWV ecology, whereby a definitive vertebrate reservoir host for POWV remains undefined, is our discovery that interspecies tick co-feeding transmission of POWV can indeed occur on a nonviremic host. Together, these data suggest that natural transmission cycles of POWV could involve a combination of multiple tick species and nonviremic co-feeding transmission, with or without a vertebrate reservoir host. Forms of vertical transmission of POWV (e.g., transovarial and transstadial transmission) have also been demonstrated in laboratory-maintained ticks [54], and recently in field-collected ticks [55]. To date, the relative importance of horizontal, vertical, and co-feeding transmission to the maintenance of POWV in nature is unknown. Therefore, future studies delineating the efficiency of these various routes of transmission (e.g., co-feeding transmission, transovarial transmission) will be critical toward improving our understanding of POWV ecology.

## Conclusions

Our findings demonstrate that under laboratory conditions, POWV II RNA can be transmitted from infected *I. scapularis* to naïve *H. longicornis* via co-feeding transmission, both in the presence and absence of host viremia. Results from this study suggest that nonviremic co-feeding transmission could play a role in the maintenance of POWV in nature; however, future laboratory, field, and modeling studies are needed to better understand POWV persistence and transmission in tick populations.

### Supplementary Information

Supplementary Material 1: **Figure 1**: Detection of POWV II RNA in donor ***I. scapularis*** females. POWV II RNA was detected in donor *I. scapularis *females by q-RT-PCR. Viral RNA was detected in 100% of virus-injected donor* I. scapularis *used in the co-feeding experiments. Viral RNA quantities are expressed as the number of NS5 gene fragment copies per ng of RNA after normalization to a standard curve. Note: The donor females were collected at varying stages of feeding (i.e., some were partially fed and others were fully engorged). Each cohort of donor females was maintained in ACL-3 facilities for ~ 1–4 weeks prior to processing and viral RNA extraction.

## Data Availability

All data supporting the conclusions of this study are provided within the manuscript or supplementary information file.

## Abbreviations

ABSL-2: Animal biosafety level 2
BSL-3: Biosafety level 3
ABSL-3: Animal biosafety level 3
ACL-3: Arthropod containment level 3
POWV II: Powassan virus lineage II
RNA: Ribonucleic acid
q-RT-PCR: Quantitative reverse transcription polymerase chain reaction
d.p.a.: Days post-attachment (of the donor *I. scapularis* female)
TBEV: Tick-borne encephalitis virus
