# Supplementary figures and images for: Interspecies co-feeding transmission of Powassan virus between a native tick, Ixodes scapularis, and the invasive East Asian tick, Haemaphysalis longicornis
Source: Parasit Vectors. 2024 Jun 15;17:259. doi: 10.1186/s13071-024-06335-0 (PMC11180395; doi:10.1186/s13071-024-06335-0)

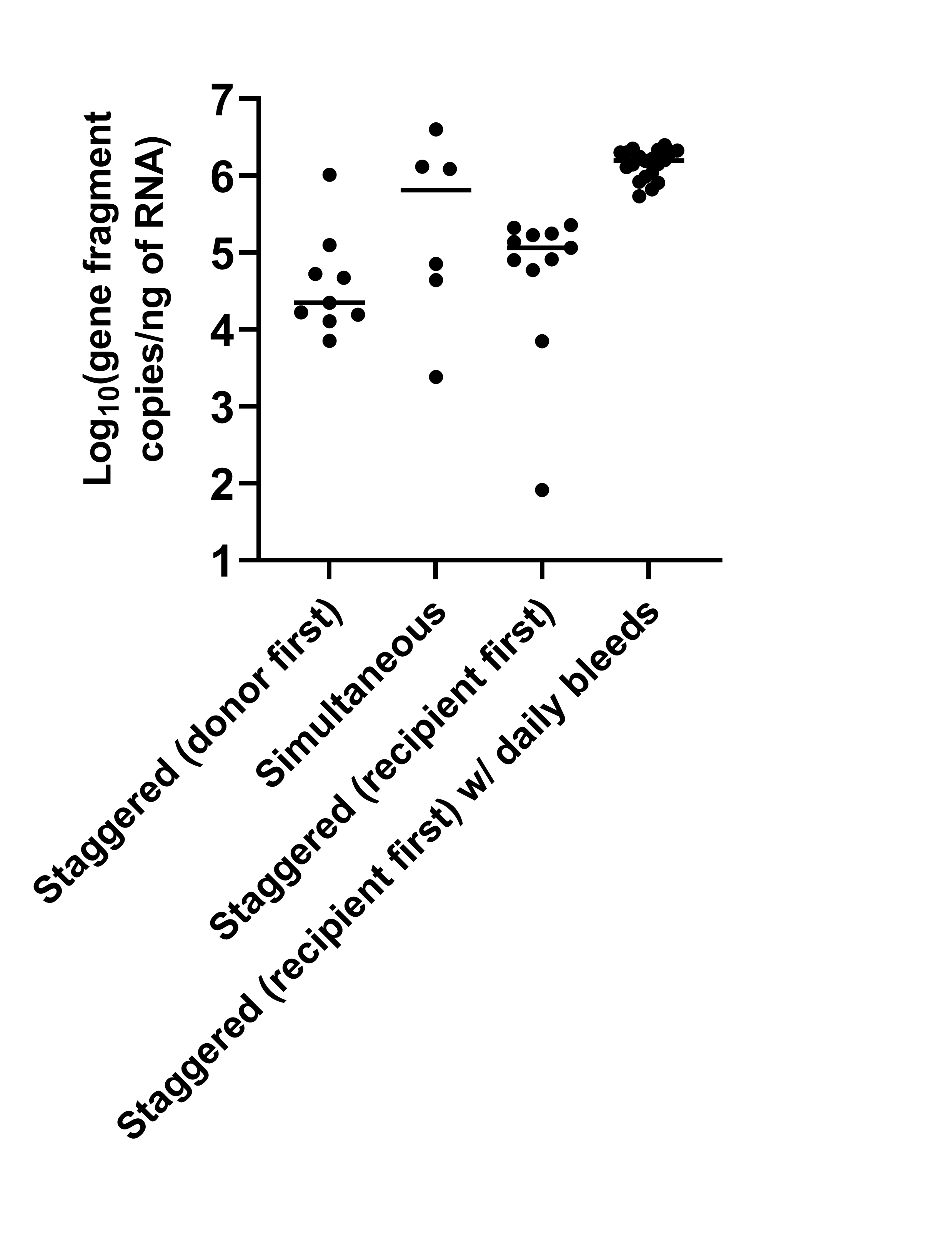

Supplement: Supplementary file 1 — Supplementary Material 1: Figure 1: Detection of POWV II RNA in donor I. scapularis females. POWV II RNA was detected in donor I. scapularis females by q-RT-PCR. Viral RNA was detected in 100% of virus-injected donor I. scapularis used in the co-feeding experiments. Viral RNA quantities are expressed as the number of NS5 gene fragment copies per ng of RNA after normalization to a standard curve. Note: The donor females were collected at varying stages of feeding (i.e., some were partially fed and others were fully engorged). Each cohort of donor females was maintained in ACL-3 facilities for ~ 1–4 weeks prior to processing and viral RNA extraction. [file 13071_2024_6335_MOESM1_ESM.tif]
